# A Scoping Review on Progression Towards Freedom from Peste des Petits Ruminants (PPR) and the Role of the PPR Monitoring and Assessment Tool (PMAT)

**DOI:** 10.3390/v17040563

**Published:** 2025-04-14

**Authors:** Dinara Imanbayeva, Maria Sol Pérez Aguirreburualde, Whitney Knauer, Azimkhan Tegzhanov, Valeriia Yustyniuk, Jonathan Arzt, Andres Perez, Felix Njeumi, Satya Parida

**Affiliations:** 1 Center for Animal Health and Food Safety, University of Minnesota, Saint Paul, MN 55108, USA; mperezag@umn.edu (M.S.P.A.); yusty002@umn.edu (V.Y.); aperez@umn.edu (A.P.); 2 Department of Veterinary Population Medicine, University of Minnesota, Saint Paul, MN 55108, USA; knaue020@umn.edu; 3 National Veterinary Reference Center, Astana 010000, Kazakhstan; azimkhan8615@gmail.com; 4 US Department of Agriculture (USDA), Agricultural Research Service (ARS), Beltsville, MD 20705, USA; jonathan.arzt@usda.gov; 5 Food and Agriculture Organization of the United Nations (FAO), Viale delle Terme di Caracalla, 00153 Rome, Italy; felix.njeumi@fao.org (F.N.); satya.parida@fao.org (S.P.)

**Keywords:** PPR Global Control and Eradication Strategy (GCES), Global Framework for the Progressive Control of Transboundary Animal Diseases (GF-TADs), Progressive Stepwise Approach, PPR eradication, PPR control, PPR-endemic countries, transboundary animal diseases

## Abstract

Peste des Petits Ruminants (PPR) is a highly contagious viral disease of small ruminants that severely threatens rural livelihoods and global food security. Under the Global Framework for the Progressive Control of Transboundary Animal Diseases (GF-TADs), the international animal health community has set the ambitious goal of eradicating PPR by 2030. However, significant disparities persist in the progression of PPR control across regions. This scoping review assesses the setbacks, deviations, and progress of 42 countries in Eastern, Western, and Northern Africa, as well as West Eurasia, toward achieving official freedom-from-PPR status. Progress was evaluated across key areas using the stepwise PPR Global Control and Eradication Strategy (GCES) approach and the PPR Monitoring and Assessment Tool (PMAT). The eligibility criteria included PubMed peer-reviewed studies, FAO/WOAH reports, presentations, guidelines, and country/region-specific PPR control plans from 2014 through 2024. The data are generated using qualitative and quantitative analyses, including spatial mapping and GCES stepwise progress evaluation. The findings reveal that many (31%) countries in the assessed regions remain in Stage 1 of the Progressive Stepwise Approach, whereas 59.5% have reached Stages 2 and 3, and only 4.8% are in Stage 4. Countries in Western Eurasia have achieved significant progress towards PPR control, with countries achieving PPR-free status, whereas, compared to Eastern and Northern Africa, the Western African region remains in the early control stages due to infrastructure gaps and resource constraints. Additionally, the recent suspension of PPR-free status in Romania, Greece and Hungary following disease emergence underscored vulnerabilities in historically free countries. The analysis results reiterate the critical role of regional collaboration, surveillance tools, and the integration of wildlife monitoring in advancing PPR control. These insights provide actionable pathways to addressing persistent barriers, highlighting the importance of adaptable, evidence-based approaches in achieving the global goal of PPR eradication by 2030.

## 1. Introduction

Peste des Petits Ruminants (PPR) is a high-priority disease for GF-TADs, as it is a highly contagious and fatal viral disease that affects small ruminants, posing a significant threat to food security, particularly in the rural communities reliant on small ruminants for livelihoods across 70 countries in Africa, the Middle East, and Asia [1,2]. PPR is a transboundary animal disease (TAD) with a substantially adverse socio-economic impact on affected countries due to the high morbidity and mortality of livestock populations [3]. The effect of the disease on rural communities’ livelihoods can be devastating, with annual losses estimated at USD 1.5 to 2 billion in over 70 affected countries across Africa, the Middle East, and parts of Asia [4]. With morbidity rates reaching up to 90% and mortality rates as high as 70%, PPR has devastating socio-economic impacts on pastoralist and agrarian communities [4,5,6,7].

PPR is caused by a *Morbillivirus* genus family of the *Paramyxoviridae* family and is generically referred to as the PPR virus (PPRV). The PPRV is an enveloped virus with a single-stranded, negative-sense RNA genome [8]. Four different PPRV lineages (I–IV) can be differentiated based on a portion of the N and F genes [9]. Historically, lineages I–II have been primarily detected in West Africa, whereas lineage III has circulated in the Middle East and Eastern Africa. After its likely origin in Western Africa, lineage IV spread eastward and became the predominant lineage across Asia; it then re-emerged in Africa, where it now seems to be the most prevalent lineage as well [10,11].

The availability of practical diagnostic tools and vaccines is a cornerstone for controlling and eradicating PPR. Rapid field diagnostic tests, such as immunochromatographic test strips and laboratory-based methods like ELISA and PCR, enable timely disease detection and monitoring. Similarly, live attenuated vaccines, such as those derived from the PPRV strains Nigeria 75/1 and Sungri 96, provide lifelong immunity against all known strains of PPR. The availability of vaccines with high thermal stability and a long shelf life to address logistical challenges is crucial. These targeted interventions will support countries to reduce the spread of the disease and contribute to global eradication efforts [12].

Because of PPR’s negative economic and financial impact on affected regions, it is crucial to establish an adequate disease control strategy following international recommendations that can prevent the disease’s further spread and alleviate its burden on people’s livelihoods. Additionally, TAD outbreaks often result in trade restrictions posed by trade partners and neighboring countries seeking to protect their territories from disease introduction or occurrence. These measures are guided by Section 5 of the Terrestrial Animal Health Code of the World Organization for Animal Health (WOAH), which outlines trade measures, import/export procedures, and veterinary certification [13]. To mitigate these risks, countries invest in preventive measures, such as vaccines and biosecurity protocols, alongside surveillance and diagnosis to enable the early detection of potential outbreaks. Therefore, good-quality data, such as real-time outbreak reports, genomic analyses, and real-time outbreak monitoring, are essential to prepare better and rapidly respond to known and emerging threats to livestock. Strong surveillance systems represent the cornerstone to providing decision-makers with adequate information to implement disease control programs, which should ideally be implemented at a regional level due to the transboundary nature of PPR.

In 2015, the Food and Agriculture Organization of the United Nations (FAO), the WOAH, and other partners launched the PPR Global Control and Eradication Strategy (GCES) in Abidjan, Côte d’Ivoire, aiming for the worldwide eradication of PPR by 2030 [12]. Building upon the success of rinderpest eradication, global initiatives for PPR elimination highlight similar challenges and opportunities for transboundary coordination [14]. The FAO/WOAH PPR Secretariat was established in 2016 to guide the implementation of the GCES. Since 2013, WOAH member countries have been eligible to apply for official recognition of their PPR-free status. By 2014, 48 countries were officially confirmed as free from PPR, with that number rising to 56 countries and one region in Namibia by 2018, and further expanded to include Russia and Lesotho by 2020 [1]. In 2024, Azerbaijan joined the list of free countries. Today, 60 countries are recognized as free from PPR, plus a free zone in Namibia. When this manuscript was written in December 2024, Romania and Greece had their PPR-free status suspended due to a PPR outbreak registered early in the year, followed by a PPR outbreak reported in Hungary in January 2025 with the disease-free status suspended [15,16,17].

The GCES follows a sequential, technical pathway for countries to progress from PPR control to complete eradication, structured across four stages: assessment, control, eradication, and post-eradication. The PPR Monitoring and Assessment Tool (PMAT) was developed as a companion to the GCES, providing countries with guidance on their current stage and pathways for advancement. PMAT, as a self-assessment tool, evaluates progress based on five technical elements outlined in the GCES: the diagnostics, surveillance, prevention, and control of PPR, a legal framework, and stakeholder involvement. An analysis of PMAT reports from 32 countries revealed that the most significant challenges lay within the “prevention and control” element, accounting for 46% of the issues identified [1].

To achieve PPR eradication, addressing one of the critical challenges of the need for alignment between national policies and the broader GCES objectives is evident. Many countries face resource constraints, political instability, and limited veterinary infrastructure, which hinder their ability to implement effective control measures [12,18,19].

This scoping review aimed to assess the multiregional evolution of PPR within the context of mapping the progress, challenges, and regional trends in the control and eradication of PPR, focusing on applying the stepwise GCES approach and PMAT framework.

By examining recent studies and international and regional organizations reports, as well as WOAH’s annual, six-monthly, and immediate notification reports, this review provides insights into how the stepwise GCES approach and PMAT framework have shaped PPR control, highlighting progress and remaining barriers to disease eradication goals by 2030. By focusing on the Eastern, Western, and Northern Africa regions and Western Eurasia, this scoping review highlights the evolution of PPR distribution and the current state of control efforts, particularly as they relate to cross-border spread and the emerging threat to the PPR-free countries in the regions. To contribute to the global effort on PPR eradication, this review advocates for the importance of regional cooperation, coordinated response strategies, and adaptable control measures by synthesizing recent findings, outbreak data, and analysis of key frameworks, contributing to global efforts toward PPR eradication.

## 2. Methodology

This scoping review followed the PRISMA-ScR (Preferred Reporting Items for Systematic Reviews and Meta-Analyses Extension for Scoping Reviews) framework [20]. No statistical meta-analysis was performed, as the objective of the scoping review was to map existing discrepancies and deviations from the GCES framework.

### 2.1. Search Strategy

A comprehensive literature search was conducted, drawing from various sources, including PubMed, FAO, and WOAH repositories, websites of regional organizations (e.g., AU-IBAR), and gray literature. The search focused on studies and reports published between January 2014 and December 2024. Our search terms were in English, as the FAO, WOAH, and GCES guidelines are primarily developed and disseminated in this language. The AU-IBAR report was produced in French and used to extract the PMAT self-evaluation results. The primary database searched was PubMed. Boolean terms (AND and OR) were used to combine the following keywords: “Peste des petits”, “PPR”, “eradication”, “control”, “monitoring”, “veterinary”, and “livestock”. Region-specific keywords (e.g., “PPR Uganda”, “PPR Kyrgyzstan”, and “PPR North Africa”) were included to ensure the inclusion of geographically relevant data for a multiregional comparison of PPR control efforts (Table 1). This search identified 618 records, with duplicates (n = 2) removed. Gray-literature searches were conducted using the official websites of the FAO and WOAH. Additional reports were sourced from the PPR Secretariat and AU-IBAR. These included regional roadmaps, official guidelines, outbreak notifications, and disease control updates. A total of 60 documents out of 119 identified were included in the review, comprising 31 peer-reviewed papers from PubMed, 18 organizational reports, three presentations, and eight other studies. The inclusion criteria were based on relevance to this scoping review objectives, focusing on the stepwise approach and completeness of the information provided.

Additional searches were conducted using ResearchGate and Google Scholar to identify relevant academic gray literature, including dissertations, theses, and unpublished reports. To complement this, we examined the reference lists of included studies and reports to identify additional resources through citation chasing.

The scope was limited to studies and reports addressing PPR control and eradication within the GCES and the PMAT framework. The scope included quantitative studies (e.g., surveillance outcomes and outbreak data) and qualitative analyses (e.g., lessons learned and regional cooperation). Review articles and reports focusing solely on unrelated diseases or regions were excluded.

### 2.2. Study Eligibility

A total of 119 studies were identified. Following the application of predefined eligibility criteria, 60 studies were included in the final review due to their focus on interventions or programs aimed at controlling and eradicating PPR in small ruminants. Eligible sources provided evidence relevant to the GCES framework, including diagnostics, surveillance, prevention, legal frameworks, and stakeholder engagement. Both quantitative and qualitative studies were included, regardless of the study design. Selected studies prioritized reports on country or region-specific data, particularly in Africa (Eastern, Western, and Northern) and Western Eurasia. English-language sources published between 2014 and 2025 and one regional report in French were included. The remaining 82 reports were excluded for the following reasons: (i) irrelevance to PPR control strategies (e.g., studies solely focusing on vaccine development, epidemiological modeling, or molecular analysis without direct relevance to the Progressive Stepwise Approach), (ii) duplication across sources (e.g., One Health initiatives without direct relevance to PPR), (iii) regions not related to this scoping review focus, and (iii) insufficient methodological details to support inclusion. Although vaccine development and modeling studies contribute to broader PPR research, they were outside the scope of this review, which focuses on evaluating national progress towards eradication using the GCES framework and PMAT assessments.

### 2.3. Record Screening and Data Extraction

Titles and abstracts identified through PubMed and other database searches were imported into Zotero for deduplication. A manual review was conducted to identify and remove duplicate records (n = 2). The remaining records were screened for relevance via titles and abstracts’ revisions against the eligibility criteria. Potentially relevant records were retrieved and assessed independently to confirm eligibility.

A data extraction table was developed and piloted to ensure consistency and reliability. Key information extracted included authors’ names, year of publication, region or country of focus, relevance to the GCES framework, and a summary of findings. All data were independently extracted and cross-checked for accuracy by the primary researcher.

### 2.4. Data Synthesis

Data were synthesized using a narrative approach, focusing on thematic patterns and trends related to PPR control and eradication. Key themes included progress along the GCES stepwise framework, veterinary capacities, outbreak management, and challenges in achieving PPR eradication by 2030. Regional and country-specific findings were compared to highlight variations in progress and barriers to implementation. Quantitative data were supplemented with spatial analysis to map high-risk zones and visualize progress along the GCES framework. Data were stratified by region (e.g., Western Eurasia and Western Africa) to align with FAO/WOAH classifications and facilitate regional comparisons of progress and challenges.

### 2.5. Bias Assessment

Methodological quality and potential bias were assessed for all included records using a tailored evaluation framework based on relevance, completeness, and alignment with the GCES framework. Organizational reports, guidelines, and gray literature were reviewed for credibility and authority, while peer-reviewed studies were assessed for methodological rigor. Studies were retained regardless of methodological quality to ensure inclusivity and a comprehensive scoping review of all available evidence consistent with the PRISMA-ScR guidelines [20,21].

## 3. Results

### 3.1. Screening Results

**Figure 1:**
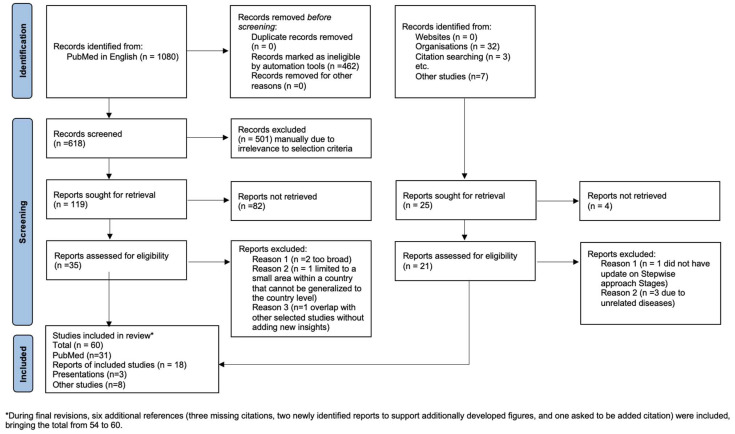
PRISMA flow diagram.

One thousand and eighty database records and forty-four additional records were identified (Figure 1). After deduplication, screening, and full-text assessments, 60 studies were included.

### 3.2. Characteristics of Included Studies

**Figure 2:**
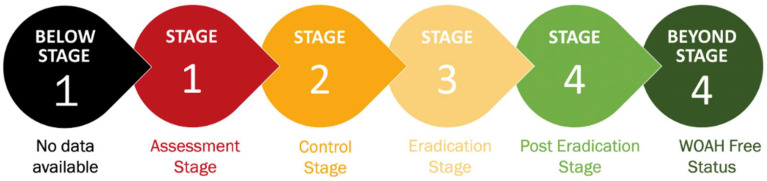
The Progressive Stepwise Approach for the prevention and control of PPR.

**Figure 3:**
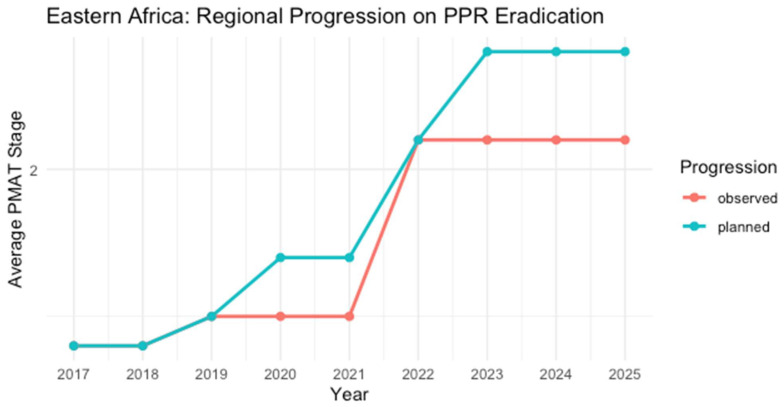
Average planned (blue line) vs. observed (red line) PPR stages across Eastern Africa from 2017 to 2025 data were aggregated from PMAT self-assessments (planned) and WAHIS outbreak reports (observed), together with the latest PPR roadmap reports [26,29,30]. A noticeable gap suggests that real-world progression is lagging behind the intended eradication roadmap in most years.

**Figure 4:**
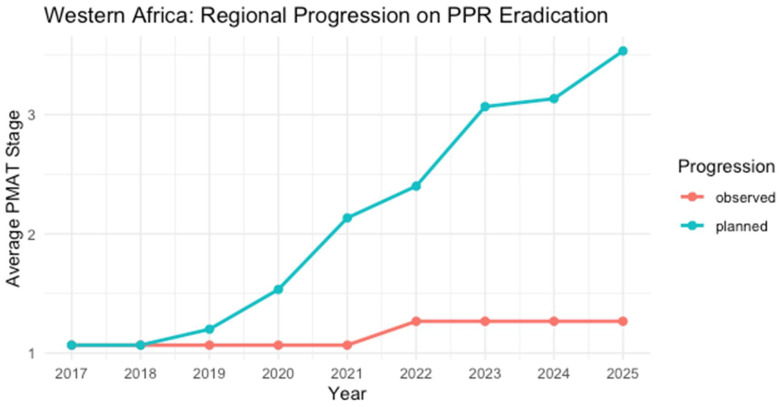
While the planned (blue line) progression towards PPR eradication envisions a rapid advancement toward higher stages in Western Africa (2017–2025), the observed (red line) data remain at lower levels, indicating that outbreaks and resource constraints have stalled progression in many countries of the region [26,37].

**Figure 5:**
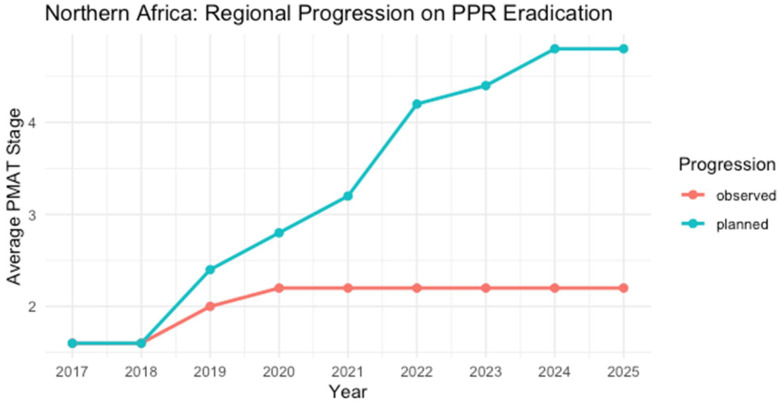
Mean planned (blue line) vs. observed (red line) PPR stages for Northern African countries, spanning 2017 to 2025. Although some nations demonstrate robust veterinary infrastructure and vaccination efforts, recent outbreaks have hindered alignment with the original roadmap, causing a persistent gap between planned and observed trajectories [26,40,45,46,47].

**Figure 6:**
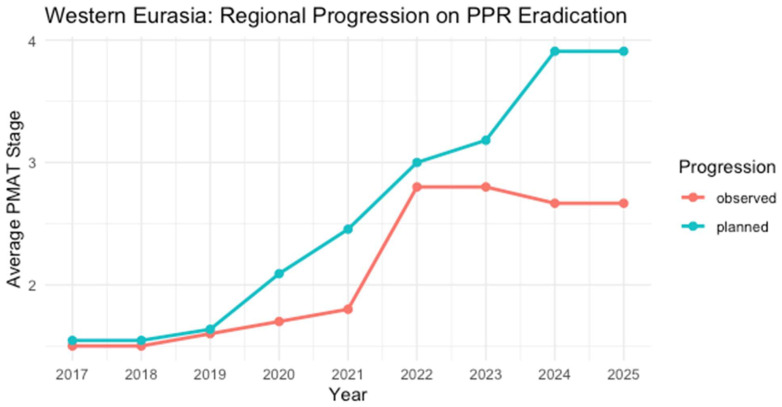
A single chart showing the upward trend in the planned (blue line) that contrasts with the observed (red line) in progression towards PPR eradication in Western Eurasia, 2017–2025. The real-world progression is different from the planned, reflecting challenges like cross-border livestock movement and outbreaks that delay countries’ progression to higher stages [26,50,53].

The 60 studies included in the review were from 2014 to 2024, comprising 31 peer-reviewed articles and 29 gray-literature sources, including organizational reports and presentations (Table 2). The studies spanned multiple regions, including Eastern, Western, and Northern Africa, as well as Western Eurasia, focusing on PPR control under the GCES and PMAT frameworks. Most studies examined countries in GCES Stages 1–3, addressing themes such as vaccination, surveillance, and barriers to eradication, including cross-border livestock movement and resource limitations. Study designs encompassed quantitative analyses, qualitative evaluations, and mixed methods (Table 3, Table 4, Table 5 and Table 6, Figure 2, Figure 3, Figure 4, Figure 5 and Figure 6).

### 3.3. The Global Control and Eradication Strategy (GCES) and PPR Monitoring and Assessment Tool (PMAT)

At the national level, the stepwise GCES approach is a multi-stage dedicated effort that helps decrease epidemiological risk levels and increase prevention and control. The four stages of the GCES involve assessment, control, eradication, and maintenance of PPR-free status [23]. The PMAT is a complementary tool designed to help countries measure their progress within this framework.

The GCES provides the roadmap for eradicating PPR, while the PMAT acts as the dashboard, allowing countries to monitor where they are on the roadmap and identify gaps they need to address. Based on this assessment, targeted actions such as improving vaccination coverage or strengthening surveillance can be implemented to advance to the next stage.

The stages range from Stage 1, at which the epidemiological situation is being assessed, to Stage 4, where the country can provide evidence that there is no virus circulation at the zonal or national level and is ready to apply for official WOAH recognition of PPR freedom. The strategy recognizes that situations and contexts can vary between countries. Additionally, PMAT as a self-evaluation tool may be biased when countries assess themselves; still, it is a powerful tool for identifying countries’ gaps and corresponding staging [27].

The GCES-specific goals and requirements for each stage are presented below (Figure 2) [22]:

- **Below Stage 1 (black): no data available**
- **Stage 1 (red): assessment**—countries evaluate the current PPR situation and identify high-risk areas.
- **Stage 2 (orange): control**—implement targeted vaccination and movement control measures.
- **Stage 3 (yellow): eradication**—intensified vaccination and surveillance efforts focus on reducing PPR incidence.
- **Stage 4 (green): post-eradication**—countries monitor for re-emergence and ensure PPR-free status, certified by WOAH.
- **Beyond Stage 4 (dark green): WOAH-free status.**

A comparison between PMAT staging and the GCES Blueprint indicated discrepancies in reported progress for several countries, often disrupted by unforeseen outbreaks.

Many countries, 38.1%, have progressed to Stage 2, with notable representation in Northern Africa and parts of Western Eurasia, reflecting efforts toward vaccination and initial control measures. Thirty-one percent of countries are still in Stage 1, predominantly in Western Africa and parts of Eastern Africa. Another 21.4% of countries are at Stage 3, concentrated in Western Eurasia, demonstrating advanced control systems, the containment of outbreaks, and readiness for eradication activities. Only 4.8% of countries have reached Stage 4. The progress is seen in specific areas of Western Eurasia, where Azerbaijan achieved official freedom from PPR in 2024 (2.35%). Additionally, 2.35% of countries are included in the PPR roadmap reports without a designated stage, as the country is not assessed by the PPR GCES Stepwise Approach.

### 3.4. Regional Findings

#### 3.4.1. Introduction to Regional Findings

Despite the global goal of eradicating PPR by 2030, several regions remain PPR-endemic. According to the PPR GCES, there are nine different regions divided following an epizone approach, involving the grouping of countries with similar epidemiology, which was also envisioned to promote the cooperation of neighboring countries that share epidemiological features but that may have been included in different roadmap regions [10]. Countries marked with ‘*’ in Table 3, Table 4, Table 5 and Table 6 experienced outbreaks that interrupted their planned progression along the GCES stepwise approach, as documented in official PMAT reports [29,53]

#### 3.4.2. Eastern Africa

PPR remains endemic across Eastern Africa. Contributing factors include nomadic livestock movement, uncontrolled regional trade, and limited veterinary infrastructure [14]. In Ethiopia, seroprevalence rates of 32.1% among unvaccinated animals and 68.8% in vaccinated ones were observed in the Borena Zone [33]. Sudan’s vaccination efforts have been hindered by insufficient access to veterinary services and surveillance gaps [41,42]. PMAT evaluations indicated discrepancies between planned and actual progress (Table 3).

**Table 3:** PMAT self-assessment results and the GCES outcome summary on the PPR Stepwise Approach Progression for Eastern Africa.

Country	2017	2018	2019	2020	2021	2022	2023	2024	2025	Last WAHIS ^1^ Outbreak Reported
Ethiopia	2	2	2	2	2	2	2	2	2	No reports
Kenya	2	2	2	2	2	2	2	2	2	No reports since 2007
Uganda	1	1	2	2	2	2	2	2	2	No reports since 2007
Sudan	2	2	2	2	2	3	3	3	3	No reports
Somalia	2	2	2	2	2	3	3	3	3	No reports
Burundi	1	1	1 *	1	1	2	2	2	2	May 2019
Djibouti	1	1	1	1	1	2	2	2	2	No reports
Eritrea	1	1	1	1	1	2	2	2	2	No reports
Rwanda										May 2024
South Sudan	1	1	1	1	1	2	2	2	2	No reports

^1^ World Animal Health Information System. * A PPR outbreak reported year, resulting in a setback in the stepwise progression. Stages: 1 (assessment—red), 2 (control—orange), 3 (eradication—yellow). Countries/zones not assessed via PPR-GCES are marked in gray. Color coding aligns with Figure 2.

PPR remains endemic across Eastern Africa, significantly affecting pastoral and rural communities that rely on small ruminants for their livelihoods. In Ethiopia, high seroprevalence levels are reported within pastoralist communities [31,33]. A study from the Borena Zone of the country, which is bordered on the south by Kenya, indicates seroprevalence rates of 32.1% among unvaccinated animals and up to 68.8% in vaccinated ones.

No WAHIS reports on PPR existed in Ethiopia, Sudan, Eritrea, South Sudan, Djibouti, or Somalia. Kenya and Uganda have not reported PPR to WOAH since 2007. The GCES has not assessed Rwanda, and it belongs to the list of countries or zones without an official PPR status for which PPR GCES stages have not been evaluated [27]. A comparison between PMAT self-evaluation staging and the GCES Blueprint outcomes reveals significant discrepancies, highlighting critical challenges. In several cases, countries’ self-assessments reflected optimistic plans to progress to the next stage. Yet, these were either not confirmed by the FAO/WOAH or disrupted by unforeseen PPR outbreaks [26,29].

#### 3.4.3. Western Africa

Most Western African countries (Table 4) remain at Stage 1 of the PMAT. Molecular studies indicate the circulation of lineage II as the dominant strain in Western Africa, but lineage IV has also been reported in neighboring regions [28]. Most countries, including Burkina Faso, Côte d’Ivoire, Ghana, Guinea, and Nigeria, have remained in Stage 1 over the past decade. Challenges such as inadequate diagnostic capacities, insufficient vaccination campaigns, and transboundary animal movements hinder progression [60]. A few notable exceptions, such as Senegal and Liberia, have progressed to Stage 2. Still, outbreaks have occurred during their efforts, delaying the further progression of the countries along the Progressive Stepwise Approach.

**Table 4:** Evaluation of the Progressive Stepwise Approach to PPR Control and Eradication in the Western African Region.

Country	2017	2018	2019	2020	2021	2022	2023	2024	2025	Last Outbreak Reported
Benin	1	1	1	1	1	2	2	2	2	No reports
Burkina Faso	1	1	1	1	1	1	1	1	1	No reports
Cabo Verde	1	1	1	1	1	1	1	1	1	No reports
Cô d’Ivoire	1	1	1	1	1	1	1	1	1	No reports
Gambia	1	1	1	1	1	1	1	1	1	No reports
Ghana	1	1	1	1	1	1	1	1	1	No reports
Guinea	1	1	1	1	1	1	1	1	1	No reports
Guinea-Bissau	1	1	1	1	1	1	1	1	1	No reports
Liberia	1 *	1	1	1	1	2	2	2	2	* December 2017
Mali	1	1	1	1	1	1	1	1	1	Last report in May 2005
Nigeria	1	1	1	1	1	1	1	1	1	No reports
Niger	1	1	1	1	1	1	1	1	1	No reports
Senegal	2	2	2	2	2	2	2	2	2	No reports
Sierra Leone	1	1 *	1	1	1	2	2	2	2	September 2018
Togo	1	1	1	1	1	1	1	1	1	No reports

* PPR outbreak reported year, resulting in a setback in the stepwise progression. Stages: 1 (assessment—red), 2 (control—orange). Color coding aligns with Figure 2.

#### 3.4.4. Northern Africa

Countries in Northern Africa, such as Egypt, Morocco, Tunisia, Algeria, and Libya, each face distinct circumstances in their efforts to contain the virus.

Northern African countries have more substantial veterinary infrastructure and a history of structured mass vaccination campaigns, though they still encounter outbreaks that delay progress in PPR control. PPR was first detected in Algeria in 2011, with the latest outbreak reported in December 2022 [45]. Algeria initially aimed for PPR-free status by 2023 under the 2019 Regional Roadmap. Still, the outbreak in 2022 reported to WAHIS affected 143 animals, including 130 sheep and 13 goats, with 20 deaths reported and delayed Algeria’s planned progression to Stage 4. Algeria continues addressing the virus circulation situation. The WAHIS report on Libya indicated a recurrence of PPR in 2020, with confirmed outbreaks affecting 4980 susceptible animals, resulting in 407 cases and 178 deaths. Introducing new live animals was identified as the likely source of infection, delaying Libya’s progression along the stepwise approach towards freedom of PPR [49].

Morocco first encountered PPR in 2008 with a lineage IV virus strain and implemented a mass vaccination campaign from 2008 to 2011. This approach effectively prevented further cases for a prolonged period [46]. However, despite Morocco’s goal of achieving free status by 2023, a new outbreak occurring in 2022 was resolved only in October 2023, signaling the ongoing need for robust surveillance and preventive measures [29]. Tunisia reported its first cases in 2009, with a similar lineage IV strain seen in neighboring countries. Following Morocco’s intention, Tunisia also aimed to achieve free status by 2023 and has since progressed significantly under the GCES through regional collaboration, targeted vaccination, and political will. However, a recurrence of PPR in 2016 in Tunisia, with 358 cases reported among 4371 susceptible animals, resulting in 185 deaths, primarily among sheep, delayed Tunisia’s progress in the PPR stepwise approach and highlighted gaps in outbreak source identification [40].

In Egypt, PPR was officially confirmed in 2012, with annual outbreaks of over 35 cases reported between 2013 and 2017. The country has since implemented a dual strategy of passive and active surveillance, combined with a mass vaccination campaign started in 2017. Egypt’s approach, which included three years of comprehensive vaccination, followed by the vaccination of newborn lambs, showcases the importance of structured veterinary infrastructure in managing and containing outbreaks.

Goals for the Northern African countries were set up at the Roadmap for the Control and Eradication of PPR in the countries of the Arab Maghreb Union (UMA) meeting, which was held in April 2019, indicating countries’ self-assessment through PMAT stages that were refined following PPR outbreaks occurred in the region of Northern Africa (Table 5) [47]. The updated report on PPR Global Eradication Programme II & III: Blueprint has indicated countries’ progress between 2017–2022 and provided comparative information to demonstrate regional progress in GCES’s progressive stepwise approach towards freedom from PPR [26].

**Table 5:** Evaluation of the Progressive Stepwise Approach Implementation in the Northern Africa Region in Achieving PPR Control and Eradication Goals.

Country	2017	2018	2019	2020	2021	2022	2023	2024	2025	Last Outbreak Reported
Algeria	2	2	3	3	3	3 *	3	3	3	December 2022
Libya	1	1	1 *	1 *	1	1	1	1	1	May 2019, October 2020
Morocco	2	2	3	3	3	3 *	3	3	3	October 2023
Mauritania	1	1	1	2	2	2	2	2	2	No WAHIS reports
Tunisia	2	2	2	2	2	2	2	2	2	* September 2016
Egypt	1 *	1	1	1	1	2	2	2	2	No outbreaks have been reported since May 2013 in WAHIS ^1^. FAO ^2^ documented no cases since 2017

* PPR outbreak reported year, resulting in a setback in the stepwise progression. ^1^ World Animal Health Information System. ^2^ Food and Agriculture Organization of the United Nations. Stages: 1 (assessment—red), 2 (control—orange), 3 (eradication—yellow). Color coding aligns with Figure 2.

#### 3.4.5. Western Eurasia

PPR presents a significant risk to the free countries in the European region due to their geographic proximity to the endemic areas of Transcaucasia, Middle East, and Central Asia, and the extensive cross-border movement of livestock is another risk of disease introduction. Western Eurasia is at various stages along the Progressive Stepwise Approach to PPR eradication [54]. While some nations have implemented robust control measures, the region faces challenges such as limited vaccination coverage, variable veterinary infrastructure, and transboundary livestock movement that facilitate the spread of PPR.

In 2024, Azerbaijan was officially declared free of PPR [51]. Georgia experienced a PPR outbreak in the spring of 2024 despite its effort to stop vaccination in the country from progressing along the progressive stepwise approach to official freedom.

The recent outbreaks in European Union (EU) countries such as Romania, Greece, and Hungary have highlighted the vulnerability of the PPR-free countries [15,16,17]. Both countries have had their free status suspended following outbreaks in 2022 and 2023, highlighting the critical need for robust surveillance, coordinated vaccination, and effective buffer zone strategies [17]. For example, a recent risk assessment study in Kazakhstan emphasized the high-risk zones in the eastern and southern regions, where trade routes and high-density livestock populations could serve as potential entry points for PPR [10,57]. Countries like Kazakhstan, Kyrgyzstan, and Uzbekistan are at elevated risk due to extensive cross-border livestock movement and trade with neighboring endemic regions, such as China and the Middle East [10,56].

Meanwhile, Kyrgyzstan, which has historically not reported PPR, benefits from natural geographical barriers, such as mountainous regions, that can slow disease spread. However, their livestock sectors are highly integrated with those of neighboring countries through informal and formal trade routes.

**Table 6:** Evaluation of the Progressive Stepwise Approach Implementation in Western Eurasia in Achieving PPR Control and Eradication Goals.

Country	2017	2018	2019	2020	2021	2022	2023	2024	2025	Last Outbreak Reported
Armenia	4	4	4	4	4	4	4	4	4	Never reported
Azerbaijan	1	1	1	1	1	4	4	Free	Free	Never reported
Georgia	1	1	2	2	3	3	3 *	3	3	May 2024
Iran	1	1	1	2	2	3	3 *	3	3	No reports
Kazakhstan	2	2	2	2	2	3	3 *	3	3	No reports
Kyrgyzstan	1 *	1	1	1	1	3	3 *	3	3	No reports
Russian Federation				Free	Free	Free	Free	Free	Free	No reports
Tajikistan	1	1	1 *	1 *	1	1	1	1	1	September 2015
Türkiye	2	2	2	2	2	3	3 *	3	3	February 2024
Turkmenistan	1	1	1	1	1	2	2	2	2	No reports
Uzbekistan	1	1	1	1	1	2	2	2	2	No reports

* PPR outbreak reported year, resulting in a setback in the stepwise progression. Stages: 1 (assessment—red), 2 (control—orange), 3 (eradication—yellow), 4 (post-eradication—green), ‘Free’ (dark-green). Color coding aligns with Figure 2.

Armenia has never reported PPR, and the country is considered historically free from PPR; it is not a part of the Progressive Stepwise Approach and, therefore, may advance to Stage 4 to collect evidence of the absence of virus circulation to apply to WOAH for freedom of PPR. Azerbaijan, a country that has never reported PPR, has applied this approach to collecting the evidence and applied it to WOAH for freedom of PPR. The PPR-free status was obtained by Azerbaijan in 2024 [25,53].

**Figure 7:**
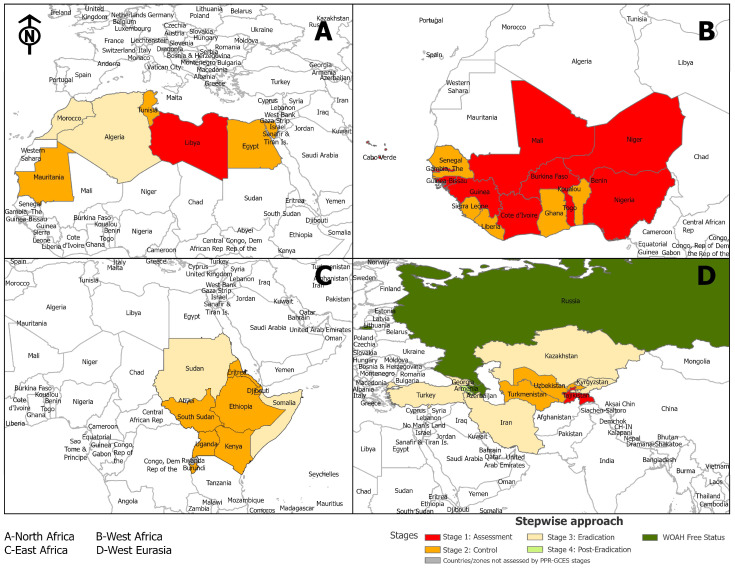
Regional progression in the PPR Stepwise Approach Towards Eradication Across Endemic Regions.

The PPR Progressive Stepwise Approach evaluation indicates the leadership of Western Eurasia in PPR control efforts (Figure 7), with advanced veterinary systems and robust surveillance enabling several countries to approach freedom from the disease (Stages 3–4).

## 4. Discussion

### 4.1. Animal Movement and Trade Impact

Pastoral mobility and illegal regional trade continue to hinder PPR control efforts. High seroprevalence in Eastern and Western African regions indicates the role of transboundary livestock movement in sustaining virus circulation [11,14,31,34,35,36,38,39,43,48]. Similar patterns have been observed in Western Eurasia, where seasonal transhumance increases the risk of PPR introduction from endemic areas such as the Middle East and China. Countries with more natural geographical barriers (e.g., Kyrgyzstan’s mountainous terrain) may see slower disease spread, but the informal livestock trade is still a threat to disease control [10,55]. A comparable risk exists in Southeast Asia, where livestock trade networks contribute to continued virus circulation and present challenges for eradication efforts [1,56]

A crucial example of livestock movement-associated outbreaks is the recent introduction of the PPR in Romania, Greece, and Hungary, where the virus was linked to cross-border transmission, suspending their free status [15,16,17]. Romania’s approach, focusing on targeted vaccination in high-risk areas and strengthened monitoring, highlights the need for cross-border cooperation and coordinated surveillance strategies [15].

### 4.2. Veterinary Infrastructure and Disease Control Challenges

Limited veterinary infrastructure remains a significant barrier to PPR eradication in multiple regions. Despite ongoing vaccination efforts in Sudan, insufficient veterinary services and limited surveillance prevent effective control. The lack of formal coordination among neighboring countries exacerbates the issue, allowing cross-border transmission to persist [41,42]. However, a structured veterinary system in Egypt has contributed to lower PPR prevalence than in neighboring nations. Egypt’s border surveillance measures serve as a model for regional containment strategies [44,48].

Countries such as Morocco, Tunisia, and Algeria have relatively advanced veterinary networks in Northern Africa, but political instability has disrupted vaccination programs, delaying progress toward PPR-free status [29]. Similarly, in Western Eurasia, variable veterinary infrastructure across countries like Georgia, Kazakhstan, Kyrgyzstan, and Uzbekistan affects their ability to implement effective surveillance and vaccination programs [10,52].

### 4.3. Vaccination Challenges

Vaccination is a key component of PPR control, but several challenges undermine its effectiveness. In Eastern Africa, Ethiopia’s Borena Zone recorded seroprevalence rates of 32.1% among unvaccinated animals and up to 68.8% in vaccinated ones, suggesting a mix of vaccination-induced and natural immunity. However, the lack of diagnostic tools to differentiate between vaccinated and naturally infected animals complicates disease monitoring [33]. DIVA (Differentiating Infected from Vaccinated Animals) tests could address this issue by distinguishing between vaccine-derived immunity and actual disease exposure. Efforts to improve vaccine coverage are constrained by the nomadic lifestyle of pastoralists and limited veterinary resources [33]. Political instability further exacerbates these issues, complicating efforts to implement and sustain vaccination programs in regions with limited veterinary services [32,33]

A similar trend is observed in Western Eurasia, where Kazakhstan and Kyrgyzstan struggle to maintain high coverage due to cross-border livestock movement [10]. Coordinated regional strategies, such as synchronized vaccination campaigns, could mitigate virus circulation in high-risk areas.

### 4.4. Regional Differences in PPR Control Progress

Given that PPR is a transboundary disease, regional and international cooperation is essential in combating the disease. Lessons from Romania’s targeted vaccination efforts highlight the need for synchronized cross-border strategies to reduce disease transmission [15]. The Transcaucasian region is strategically crucial, occupying a small territory on the border of Eastern Europe, Western Eurasia, and the Middle East. It is a natural bridge between these regions, playing a strategic role in terms of TADs introduction and spread into Europe. PPR and other TADs introduction to the European countries via the Transcaucasian region can pose a risk of TADs introduction to the European Union that has been previously free of PPR, causing profound implications not only in the European countries but its neighborhood, where rural communities are heavily relying on small ruminants’ production due to its historically lasting cultural, social, and economic assets [52]. The recent outbreaks in Romania, Greece, and Hungary are examples of these implications the European region faces [15,16,17]. Thus, coordinated surveillance, real-time outbreak reporting, and harmonized vaccination campaigns are critical to sustain PPR control.

Western Eurasia’s advancements in veterinary infrastructure were made possible through structured investments, while Eastern and Western Africa struggled with limited resources. To achieve PPR eradication by 2030, long-term funding commitments from governments, regional organizations, and international donors are needed to sustain vaccination programs, strengthen veterinary services, and improve diagnostics. While this scoping review focuses on PPR control efforts in African regions and Western Eurasia, similar challenges related to resource constraints, transboundary livestock movement, and gaps in surveillance, are observed in other endemic regions, including South and East Asia, and the Middle East [1,12]. A future study could further explore these regions to provide a more comprehensive global assessment of PPR eradication progress.

While the GCES framework provides a structured approach to PPR eradication, real-world factors, such as unexpected outbreaks, often lead to deviations from planned country progress.

## 5. Key Findings

Despite significant progress over the last decade under the PPR GCES, gaps in vaccination coverage, disease surveillance, and cross-border coordination exist in all regions covered here. Various endemic regions face persistent challenges that undermine eradication efforts. However, a notable limitation across all regions is the absence of clear, objective metrics or indicators to measure progress between stages. For instance, while ‘intensified vaccination’ is a common strategy, there are no standardized benchmarks such as minimum vaccination coverage thresholds or seroconversion rates to assess the effectiveness of these efforts. This lack of quantitative metrics contributes to discrepancies between countries’ self-assessments and readiness for stage progression, as seen in PMAT evaluations. Establishing measurable indicators would enable more accurate assessments and ensure alignment with eradication goals. An analysis of PMAT reports reveals that “prevention and control” remains the most challenging area, accounting for 46% of identified issues [2,4,22].

It is noted that the Western Eurasia region exhibits a mosaic of PPR statuses, with some countries like Azerbaijan and Russia achieving Stage 4 in the Progressive Stepwise Approach. In contrast, others lag in Stage 1 or 2 (e.g., Uzbekistan and Tajikistan). Several countries, such as Kazakhstan and Kyrgyzstan, have invested in targeted risk-based vaccination campaigns in high-risk areas. Despite this approach, difficulties sustaining reliable vaccination coverage due to cross-border livestock movement and insufficient post-vaccination evaluations are in place [10]. In Eastern Europe, recent outbreaks in Romania, Greece, and Hungary underscore the vulnerability of PPR-free countries, even those with robust veterinary infrastructure. These outbreaks highlight the importance of regional coordination to mitigate cross-border disease risks and emphasize the importance of contingency plans, including emergency vaccine stock availability and rapid response teams’ readiness [15,16,17]. Countries such as Turkey and Iran, where vaccination remains critical, have shown the value of structured vaccination and surveillance systems. However, these countries continue to face challenges related to the enforcement of movement restrictions and wildlife monitoring [10,54].

In Eastern Africa, pastoralist movement and limited veterinary infrastructure hinder vaccination campaigns and surveillance efforts. The high reported seroprevalence reports in Ethiopia, Kenya, and Uganda highlight ongoing virus circulation, while political instability exacerbates the challenges of sustaining vaccination programs and surveillance systems [31,36]. Additionally, nomadic populations pose a significant barrier to achieving consistent vaccination coverage. In Eastern and Western Africa, transhumance limits vaccine administration, and outbreaks often coincide with gaps in vaccination coverage. Addressing these challenges requires targeted risk-based vaccination strategies and improved outreach to mobile communities. Countries in Northern Africa, such as Algeria, Tunisia, and Morocco, have progressed in PPR control, but outbreaks continue to delay progression to PPR-free status. The epidemics in Algeria and Morocco underscore the need for robust vaccination programs and improved outbreak source identification. In Western Africa, Burkina Faso, Ghana, and Guinea face barriers such as informal livestock trade and poorly enforced movement controls. Senegal and Liberia have progressed to Stage 2 but experienced challenges due to outbreaks during their efforts.

Phylogenetic analysis has proven effective in tracing viral lineages and understanding transboundary movements. For instance, genetic studies in Western Africa identified lineage II as the dominant strain, which can guide targeted vaccination and movement control strategies in endemic regions [39]. Expanding genetic surveillance in Western Eurasia could offer similar benefits by identifying high-risk zones and informing regional collaboration.

Tailored vaccination strategies are essential for regions with nomadic pastoralism and informal livestock trade. The Black Sea Basin study highlights the importance of harmonizing regional vaccination efforts and developing joint buffer zones to mitigate transboundary risks [54]. Synchronized cross-border vaccination efforts could significantly reduce virus circulation. Similar approaches could be applied in the Transcaucasian region, where shared borders between endemic and PPR-free countries require stronger cooperation. Outbreaks among saiga antelope populations in Mongolia emphasize the role of wildlife in PPR dynamics [24,59]. Wildlife population monitoring in Western Eurasia could provide early warning signs of virus circulation and help prevent spillover into livestock populations [58].

Addressing gaps in veterinary service delivery, particularly in rural and remote areas, is critical for sustaining vaccination campaigns and border-level biosecurity enforcement. The success of National Animal Identification and Traceability Systems (NAITS) in other regions highlights its potential applicability in Africa and Western Eurasia [54]. Although regional and international cooperation is crucial, it is often challenging without sustainable international funding and political support.

While PPR control progress varies regionally, funding remains a significant determinant of success. Financial constraints hinder disease control in Eastern and Western Africa, while structured investments in Western Eurasia have contributed to progress.

An additional key finding of this review is the delineation of limitations of the current framework for evaluating progress along the PPR GCES stages, which is the absence of specific, objective metrics to validate country assessments. While PMAT provides a structured framework with semi-quantitative scoring for activities across technical elements, it relies heavily on process-based indicators (e.g., completing activities, implementing plans) rather than outcome-oriented metrics (e.g., achieving vaccination coverage thresholds or reducing outbreak frequency). Current indicators, such as disease prevalence or vaccination coverage, provide some guidance but are insufficient to capture the impact of key interventions like vaccination campaigns or enhanced surveillance. Without standardized, quantitative benchmarks such as vaccination coverage exceeding 80% or seroconversion rates post-vaccination, ensuring consistent and rigorous assessments across countries is challenging. Establishing objective metrics for each stepwise approach stage would strengthen the validity of the transition from one stage to another, guiding countries in identifying areas for targeted interventions and resource allocation that will increase accountability. Incorporating such metrics would address the gaps in the current system, ensuring that progress is measurable and aligned with eradication goals. This approach underscores the significance of developing a framework that integrates process and outcome indicators, a gap this review aims to address.

## 6. Limitations

### 6.1. Data Availability

This scoping review faced several limitations that should be acknowledged. Data availability varied across the studied regions and, therefore, may have affected the reliability of conclusions made. Most countries lacked updated or comprehensive reports on PPR outbreaks, vaccination coverage, and surveillance outcomes. Wildlife data were sparsely and inconsistently reported, particularly concerning transboundary movements and spillover risks [1]. The review was limited to regions actively implementing GCES and PMAT frameworks, primarily focusing on Eastern Africa, Western Africa, Northern Africa, and Western Eurasia. Other PPR-endemic regions’ situations that could have contributed to this review and provided valuable insights were not included in this study, potentially limiting the generalizability of the findings.

### 6.2. Methodological Challenges

A significant limitation was that some of the reports reviewed were not consistent or published in a manner that allowed consistent annual progress assessments. This lack of finalized documentation limited the ability to evaluate trends over time for certain countries [1]. Additionally, PMAT self-assessments introduced potential bias, with countries possibly overestimating or underestimating their progress toward achieving GCES milestones. This impacts the accuracy and consistency of stage-based evaluations and regional comparisons. Finally, this review relied heavily on English-language sources, so it may have excluded relevant studies or reports published in other languages. Expanding language coverage could provide a more comprehensive understanding of global efforts to promote PPR control and eradication.

## 7. Conclusions

This review has highlighted the persistent challenges and emerging opportunities in the global effort to eradicate PPR by 2030. The analysis underscores significant gaps in vaccination coverage, disease surveillance, and cross-border coordination across African regions and Western Eurasia. Regional differences in veterinary infrastructure, socio-economic contexts, and disease dynamics necessitate tailored control strategies. By leveraging genetic tools, integrating wildlife monitoring, and strengthening cross-border cooperation, countries can develop adaptive strategies tailored to their unique socio-economic and ecological contexts. Sustainable international support and evidence-based interventions will be critical for overcoming these challenges and achieving the global goal of PPR eradication. Despite these challenges, the stepwise progressive GCES approach, accompanied by PMAT, provides a valuable framework for guiding countries along the process of fulfilling the global goal of PPR eradication.

## Figures and Tables

**Table 1:** PubMed search terms for a scoping review evaluating PPR control, distribution, and the role of PMAT.

Concept	Keywords
Peste des petits ruminants	“Peste des petits ruminants” OR “PPRV” OR “PPR eradication” OR “PPR control” OR “PPR outbreaks” OR “PPR vaccination”
Regions/countries	“Western Africa” “Benin” OR “Burkina Faso” OR “Cape Verde” OR “Côte d’Ivoire” OR “Gambia” OR “Ghana” OR “Guinea” OR “Guinea-Bissau” OR “Liberia” OR “Mali” OR “Niger” OR “Nigeria” OR “Senegal” OR “Sierra Leone” OR “Togo” “Northern Africa” “Algeria” OR “Egypt” OR “Libya” OR “Mauritania” OR “Morocco” OR “Sudan” OR “Tunisia” “Eastern Africa” “Burundi” OR “Djibouti” OR “Eritrea” OR “Ethiopia” OR “Kenya” OR “Rwanda” OR “Somalia” OR “South Sudan” OR “Uganda” OR “Kazakhstan” OR “Kyrgyzstan” OR “Tajikistan” OR “Turkmenistan” OR “Uzbekistan” OR “Central Asia” OR “Africa” OR “Eastern Europe” OR “Russia” OR “Greece” OR “Romania” OR “West Eurasia” OR “Armenia” OR “Azerbaijan” OR “Georgia” OR “Turkey” OR “Iran” OR “Europe”
GCES	“diagnostics” OR “surveillance” OR “prevention” OR “vaccination” OR “legal framework” OR “stakeholder involvement” OR “veterinary capacities” OR “PMAT”

**Table 2:** Summary of included studies.

Author (Year)	Study Region	Study Focus	Key Findings	Relevance to PPR Control
Global Level				
[1] Zhao et al. (2021)	Global	Progress toward PPR eradication through vaccination	Emphasized vaccination campaigns achieving up to 70% efficacy in high-risk zones	Highlights vaccination as a critical component of the PPR eradication strategy under GCES
[3] Torres-Velez et al. (2019)	Global	Review of TADs as re-emerging threats with an emphasis on their One Health impact	Socio-economic impact of TADs, including PPR, highlighting the need for diagnostics, vaccination, and regional cooperation	Underscores PPR’s role as a significant TAD affecting small ruminants, emphasizing the importance of surveillance, vaccination, and control strategies to mitigate its impact
[4] FAO/WOAH (2015)	Global	Launch and framework of the PPR Global Control and Eradication Strategy (GCES)	A stepwise approach for PPR eradication, emphasizing vaccination, surveillance, and legal frameworks, is outlined	Provides the foundation for global coordination and guidance toward PPR eradication by 2030, including monitoring tools like PMAT
[22] Leboucq et al. (2015)	Global	PMAT for evaluating PPR control progress	A structured, evidence-based framework for assessing country progress in PPR control	Supports risk-based decision-making, regional coordination, and harmonized interventions
[2] Cameron (2019)	Global	Guerrilla strategy for PPR eradication	Proposed a short-duration, high-coverage vaccination strategy integrated with movement management and real-time surveillance	Advocates for sustainable, efficient eradication methods over long-term campaigns, emphasizing rapid response and targeted interventions
[5] FAO/WOAH (2024)	Global	Initiation of the Global Framework for the Progressive Management of Transboundary Animal Diseases (GF-TADs)	The Joint FAO-WOAH initiative aims to stop, identify, and manage transboundary animal diseases (TADs) by fostering regional partnership	Establishes a fundamental framework for international collaboration and local strengthening in the fight against TADs, such as PPR, through the alignment of resources and strategies at various levels
[6] Torsson et al. (2020)	Global, field applications	Field-adapted complete genome sequencing of PPRV using Nanopore technology	Demonstrated successful field-adapted protocol for full genome sequencing of PPRV, achieving >90% genome coverage in resource-constrained settings with portable devices	Enables rapid diagnostics and surveillance for PPR in remote areas, supporting FAO/WOAH eradication efforts by 2030
[7] FAO/WOAH (2021)	Global	Guidelines for PPR Control in Wildlife Populations	Approaches to incorporating wildlife aspects into PPR control initiatives encompass monitoring, risk evaluation, and managing outbreaks in wildlife populations	Emphasizes the importance of a multisectoral approach and tailored strategies to manage PPR at the wildlife–livestock interface for successful eradication
[9] Baron et al. (2016)	Global	Genetic characterization and molecular biology of PPRV	Described four distinct PPRV lineages (I–IV), which can be discriminated based on nucleotide variations in the N gene	Provides molecular tools for epidemiological surveillance, lineage tracking, and targeted control strategies for specific regions
[13] World Organisation for Animal Health (WOAH). Terrestrial Code Online Access (2024)	Global	Terrestrial Animal Health Code	Section 5 of the Terrestrial Code focuses on trade measures, import/export procedures, and veterinary certification to manage TAD outbreaks, mitigate trade restrictions, and protect territories	Provides guidelines for implementing disease control strategies aligned with international standards to minimize PPR spread and economic burdens while enabling trade continuity
[14] Taylor (2016)	Global	Eradication strategies for PPR, building on lessons from rinderpest eradication	Highlights the critical role of international cooperation, vaccination campaigns, and sanitary livestock movement to reduce virus spread and achieve control	Provides a strategic framework for PPR eradication efforts globally based on proven methods
[19] Jones et al. (2016)	Global	Benefit-cost analysis for the eradication of PPR	The analysis highlights strong economic returns from eradication through reduced mortality, increased production, and avoided control costs	Emphasizing the importance of targeted vaccination and international cooperation for resource allocation
[20] Tricco et al. (2018)	Global	Development of the PRISMA Extension for Scoping Reviews (PRISMA-ScR) Checklist	Offers detailed explanations and examples for each checklist item of scoping review	Ensures the methodological transparency and consistency of scoping reviews, applicable for structuring and reporting reviews in PPR-related research
[21] Page et al. (2021)	Global	Updates to PRISMA guidelines for systematic reviews	A 27-item checklist for transparent and comprehensive reporting of systematic reviews	Systematic reviews could enhance the quality and reliability of PPR-related research synthesis, aiding in evidence-based policy and control strategies
[23] Food and Agriculture Organization (FAO) (2016)	Global	Stepwise Approach for PPR Eradication	Progressive Control Pathway (PCP) as a framework for countries to achieve PPR eradication by 2030; identified stages and criteria for monitoring progress	A structured and systematic approach to guide countries in assessing their PPR control capabilities, identifying gaps, and implementing targeted measures for eradication
[24] Munir (2014)	Global	Role of small wild ruminants in PPR epidemiology	Phylogenetic analysis confirmed all PPRV isolates from wild ungulates belong to lineage IV	Highlights the uncertain role of wildlife in PPR epidemiology
[25] Lysholm (2024)	Global	WOAH guidelines for PPR control and disease status recognition	Outlined key provisions of the WOAH Terrestrial Animal Health Code related to PPR	Provides regulatory guidance for PPR control and eradication
[26] FAO & WOAH (2022)	Global	PPR Global Eradication Programme (GEP) II & III Blueprint	Provides a strategic roadmap for the global eradication of PPR by 2030	Outlines a structured, stepwise strategy for achieving global PPR eradication
[23] FAO (2015)	Global	Stepwise approach to PPR control and eradication	The PPR Global Eradication Programme (PPR GEP) outlines a four-stage stepwise approach	Provides a structured framework for countries’ progressive control and eradication of PPR
[27] Chemis, V. (2022)	Middle East, global	Implementation of a PPR Monitoring and Assessment Tool	Insights into PMAT’s role in evaluating national and regional efforts in PPR eradication	Demonstrates PMAT’s value in providing a structured framework for countries to assess their progress in PPR control, identify gaps, and implement corrective actions
[8] Parida et al. (2015)	Africa, Asia	Overview of PPR epidemiology and its impact	Detailed the socio-economic impact of PPR and the role of vaccination and diagnostics in eradication efforts	Supports prioritization of vaccination campaigns and diagnostic improvements in endemic regions
[12] Mariner et al. (2016)	Africa, Asia, Middle East	Challenges and Opportunities in PPR Eradication	Political instability, resource constraints, and weak veterinary systems hinder PPR control; emphasized the critical role of surveillance, vaccination, and stakeholder collaboration	Provides insights into aligning eradication strategies with socio-economic and political realities, ensuring feasibility and sustainability in resource-limited regions
[28] Dundon et al. (2020)	Africa	Review of molecular epidemiological data on PPR in Africa	Consolidated data from multiple African countries highlighting the shift toward lineage IV as the dominant strain	Provides a comprehensive molecular epidemiological perspective on PPRV circulation in Africa
Eastern Africa				
[29] AU-IBAR (2019)	Eastern Africa	Regional coordination and progress review on PPR control and eradication	Reviewed regional and national PPR control progress	Strengthens the case for regional collaboration, structured funding approaches, and cross-border coordination to advance PPR eradication
[30] WOAH (2019)	World Animal Health Information System (WAHIS) report	PPR outbreak follow-up report	Outbreak that started in 2018 was resolved in 2019	Burundi continues experiencing outbreaks
[31] Wendimu et al. (2024)	Central Oromia, Ethiopia	Seroprevalence associated risk factors	High seroprevalence was found in nomadic areas, necessitating tailored vaccination strategies for pastoral systems	Indicates risk factors that hinder vaccination coverage in regions with mobile livestock populations
[32] Fournié et al. (2018)	Ethiopia	Mathematical modeling of PPRV transmission	Used a dynamic transmission model to estimate PPRV spread and the required vaccination coverage for elimination in Ethiopia	Provides evidence-based vaccination strategies for PPR eradication
[33] Kumbe et al. (2024)	Ethiopia (Borena Zone)	Seroprevalence and risk factors of PPRV	A cross-sectional study found a PPR seroprevalence of 32.1% in non-vaccinated animals, 45.5% in animals with unknown vaccination history, and 68.8% in vaccinated animals	Highlights critical gaps in Ethiopia’s PPR vaccination coverage and eradication strategy
[34] Nkamwesiga et al. (2019)	Karamoja, Uganda	Identifying PPR transmission hotspots for targeting eradication interventions	Identified hotspots using phylogenetic and movement data to tailor vaccination campaigns	Demonstrates the importance of hotspot mapping for efficient use of resources
[35] Nkamwesiga et al. (2023)	Uganda	Seroprevalence and risk factors of PPRV in different production systems	Found a true seroprevalence of 27.3%, with the highest rates in pastoral (44.1%) and agropastoral (31.7%) systems	Emphasizes the need for targeted PPR control strategies, particularly in pastoralist communities
[36] Ayebazibwe et al. (2022)	Uganda	Assessment of animal health systems and coordination mechanisms for PPR control	PPR was first detected in Karamoja in 2007 and has since spread to over 50 districts; the study found that Uganda remains at Stage 2 of the PPR GCES	Highlights the need for improved veterinary governance, strengthened surveillance, and better vaccination logistics
[18] Britton et al. (2019)	Southern African Development Community (SADC) region	Progress to control and eradication of PPR, gaps in veterinary services, and surveillance in the SADC region	Gaps in vaccination, diagnostics, and surveillance systems are described	The need for regional cooperation, risk-based surveillance, and cross-border livestock trade control are emphasized
Western Africa				
[37] Tounkara (2017)	Western Africa	Regional PPR Strategy and Progression	Countries indicated planned progression along the Progressive Stepwise Approach	The event sought to synchronize national efforts and boost the collective ability to combat PPR
[11] Couacy-Hymann et al. (2023)	Burkina Faso, Côte d’Ivoire, Ghana, Guinea	Molecular characterization of PPRV strains	Identified genetic heterogeneity within Lineage IV, subdivided into distinct subclades, and highlighted transboundary animal movements as a key driver of virus spread	Reinforces the importance of cross-border collaboration and lineage-specific diagnostics for effective disease control
[38] Adeoye et al. (2022)	Nigeria	Molecular detection of PPRV in goats and sheep in Ibadan, Oyo State	Confirmed a 7.4% prevalence of PPRV in field samples; confirmed ongoing circulation of PPRV in Ibadan despite vaccination programs	Highlights the need for continuous PPR surveillance and more effective vaccination strategies
[39] Tounkara et al. (2018)	Niger and Western Africa	Genetic characterization of PPR in Niger	Demonstrates transboundary spread of the Asian virus lineage in West Africa	Provides evidence for the need for regional coordination in genetic surveillance and vaccination
Northern Africa				
[40] FAO & WOAH (2019)	Northern Africa	The PPR roadmap meeting in the Maghreb region (Union du Maghreb Arabe, UMA)	Countries indicated planned progression along the Progressive Stepwise Approach	The event sought to synchronize national efforts and boost the collective ability to combat PPR
[41] Intisar et al. (2017)	Sudan (Northern Africa)	Seroprevalence and molecular detection of PPRV in domestic ruminants and camels	Investigated the prevalence of PPR in sheep, goats, cattle, and camels in Sudan from 2008 to 2012	Provides strong evidence of widespread PPRV circulation in Sudan
[42] Ali et al. (2023)	Central–Western Sudan	PPRV antibodies prevalence in livestock populations	High antibody prevalence indicates ongoing virus circulation	Reinforces the role of serological studies in monitoring and targeting eradication strategies
[43] Ali et al. (2019)	Sudan	Serological investigations of PPR in cattle populations	A serosurveillance study conducted from 2015 to 2016 found a 42.0% prevalence of PPRV antibodies among 1000 cattle in five Sudanese states	Reinforces the importance of cross-species monitoring for effective PPR eradication
[44] Soltan et al. (2014)	Egypt	Molecular detection and genetic characterization of PPRV in Ismailia province	Confirmed the emergence of PPRV in Egypt despite the country previously declared PPR-free	Demonstrates the importance of continuous surveillance to detect emerging PPR outbreaks
[45] WOAH (2022)	Algeria	PPR outbreak follow-up report	Recurrence of PPR	Insights into the current PPR situation in Algeria
[46] WOAH (2016)	Tunisia	PPR outbreak follow-up report	The report provides detailed information on the latest PPR outbreaks in Tunisia	Highlights Tunisia’s efforts to control PPR
[47] WOAH (2023)	Morocco	PPR outbreak follow-up report	The report provides detailed information on the latest PPR outbreaks and its resolution in 2023	Highlights ongoing outbreaks registration
[48] FAO (2024)	Egypt	Overview of PPR epidemiological situation and control measures in Egypt	PPRV was confirmed in a sheep farm in 2012; between 2013 and 2017, at least 35 outbreaks were reported annually	Overview of Egypt’s PPR status and control strategies
[49] WOAH (2021)	Libya	PPR outbreak follow-up report	The report provides detailed information on the latest PPR outbreaks and its resolution in 2021	Highlights ongoing outbreaks registration
Western Eurasia				
[50] WOAH (2025)	Western Eurasia	Third PPR roadmap meeting	Planned progression of countries along the Progressive Stepwise Approach	Majority of the countries were planning to be free or at Stage 4 by 2025
[15] Parida et al. (2024)	Europe (Greece, Romania)	Outbreaks and control measures for PPR reintroduction to the European Union	Linked to migratory livestock movement and trade, with control measures application	Emphasizes the importance of rapid response, strict movement controls, and enhanced surveillance
[16] WOAH (2025)	Europe (Hungary)	Suspension on PPR-free status in Hungary due to outbreak	Update on Resolution No. 25 adopted in May 2024 by the World Assembly of Delegates listing Hungary as a “PPR free country”: Hungary’s status had been suspended with effect on 23 January 2025	Emphasizes the importance of rapid response, strict movement controls, and enhanced surveillance
[17] WOAH (2024)	Europe (Greece, Romania)	First detection of PPR in Greece and Romania	In July 2024, PPR was detected for the first time in Greece and Romania, both previously free of the disease	Highlights the importance of rapid detection and implementation of control measures in regions previously free from PPR
[51] WOAH (2024)	Azerbaijan	Official recognition of PPR-free status	In 2024, WOAH declared Azerbaijan free of PPR	Provides a model for other countries aiming to achieve PPR-free status
[52] Chenais et al. (2021)	Georgia	Participatory epidemiology study on small ruminant health and PPR perception	PPR was not identified as a priority disease by the participants, and no historical unreported outbreaks were detected	Suggests that PPR interventions should integrate broader livestock health management approaches
[53] FAO & WOAH (2023)	Economic Cooperation Organization (ECO) countries	Regional meeting and PPR Blueprint consultation	Evaluated national PPR eradication progress, identified challenges, and aligned country strategies with the PPR Global Eradication Program	Reinforces the need for harmonized policies, surveillance strategies, and funding mechanisms
[54] Arede et al. (2024)	Black Sea Basin	Examination of factors influencing ruminant disease dynamics, including transboundary animal diseases	Surveillance gaps, trade movements, and disease reporting inconsistencies in the Black Sea Basin	Importance of regional cooperation, robust surveillance, and policy frameworks
[10] Legnardi et al. (2022)	Western Eurasia	Epidemiological situation and control activities after Phase 1 of the PPR Global Eradication Programme	Documented the success of phased vaccination and surveillance efforts, reducing virus transmission	Highlights strategic implementation of GCES steps in higher-capacity regions.
[55] Kock et al. (2015)	Kazakhstan	Detection of PPR Lineage IV	Identifies genetic lineage IV in Kazakhstan, showing the regional spread	Highlights the importance of gene surveillance for targeted vaccination strategies
[56] Gao et al. (2021)	Western China and neighboring countries	Predictive modeling for PPR spread	Develops a predictive model showing high-risk areas for PPR spread in Western China	Provides tools for risk-based control and prioritization of resources
[57] Abdrakhmanov et al. (2022)	Kazakhstan	Mapping PPR risks	Highlights high-risk areas for PPR spread due to livestock movement and trade	Guides targeted vaccination and surveillance efforts.
[58] Pruvot et al. (2020)	Mongolia	PPRV outbreak in wild ungulates and its impact on conservation efforts	Confirmed spillover of PPRV from livestock to wild species (Mongolian saiga, ibex, goitered gazelle), causing mass mortality	Highlights the importance of including wildlife in PPR eradication efforts.
[59] Benfield et al. (2021)	Mongolia	Molecular epidemiology of PPRV in Mongolian wildlife	Phylogenetic analysis confirmed that PPRV in Mongolian wildlife likely originated from livestock transmission	Demonstrates the importance of early surveillance at the livestock–wildlife interface

## Data Availability

The original contributions presented in this study are included in the article. Further inquiries can be directed to the corresponding author.

## References

[1] Zhao H., Njeumi F., Parida S., Benfield C.T.O. (2021). Progress towards Eradication of Peste Petits Ruminants through Vaccination. Viruses.

[2] Cameron A.R. (2019). Strategies for the Global Eradication of Peste Petits Ruminants: An Argument for the Use of Guerrilla Rather Than Trench Warfare. Front. Vet. Sci..

[3] Torres-Velez F., Havas K.A., Spiegel K., Brown C. (2019). Transboundary Animal Diseases as Re-Emerging Threats—Impact on One Health. Semin. Diagn. Pathol..

[4] World Organisation for Animal Health (OIE), Food and Agriculture Organization of the United Nations (FAO) (2015). The Global Strategy for the Control and Eradication of PPR.

[5] Global Framework for the Control of Transboundary Animal Diseases. https://www.gf-tads.org/.

[6] Torsson E., Kgotlele T., Misinzo G., Johansson Wensman J., Berg M., Karlsson Lindsjö O. (2020). Field-Adapted Full Genome Sequencing of Peste-Petits-Ruminants Virus Using Nanopore Sequencing. Front. Vet. Sci..

[7] World Organisation for Animal Health (OIE), Food and Agriculture Organization of the United Nations (FAO) (2021). Guidelines for the Control and Prevention of Peste Petits Ruminants (PPR) in Wildlife Populations. Peste Petits Ruminants Global Eradication Programme.

[8] Parida S., Muniraju M., Mahapatra M., Muthuchelvan D., Buczkowski H., Banyard A.C. (2015). Peste Petits Ruminants. Vet. Microbiol..

[9] Baron M.D., Diallo A., Lancelot R., Libeau G. (2016). Peste Petits Ruminants Virus. Adv. Virus Res..

[10] Legnardi M., Raizman E., Beltran-Alcrudo D., Cinardi G., Robinson T., Falzon L.C., Djomgang H.K., Okori E., Parida S., Njeumi F. (2022). Peste Petits Ruminants in Central and Eastern Asia/West Eurasia: Epidemiological Situation and Status of Control and Eradication Activities after the First Phase of the PPR Global Eradication Programme (2017–2021). Animals.

[11] Couacy-Hymann E., Berete K., Odoom T., Zerbo L.H., Mathurin K.Y., Kouakou V.K., Doumbouya M.I., Balde A., Ababio P.T., Ouoba L.B. (2023). The Spread of Peste Petits Ruminants Virus Lineage IV in West Africa. Animals.

[12] Mariner J.C., Jones B.A., Rich K.M., Thevasagayam S., Anderson J., Jeggo M., Cai Y., Peters A.R., Roeder P.L. (2016). The Opportunity To Eradicate Peste Petits Ruminants. J. Immunol..

[13] Terrestrial Animal Health Code. https://www.woah.org/en/what-we-do/standards/codes-and-manuals/terrestrial-code-online-access/.

[14] Taylor W. (2016). The Global Eradication of Peste Petits Ruminants (PPR) within 15 Years—Is This a Pipe Dream?. Trop. Anim. Health Prod..

[15] Parida S., Yusuf J., Njeumi F. (2024). Update on Peste Petits Ruminants in Europe. Vet. Rec..

[16] PPR Free Members. https://www.woah.org/en/disease/peste-des-petits-ruminants/#ui-id-5.

[17] World Organization for Animal Health First Detection of Peste Petits Ruminants (PPR) in Greece and Romania. https://www.woah.org/en/first-detection-of-peste-des-petits-ruminants-ppr-in-greece-and-romania/.

[18] Britton A., Caron A., Bedane B. (2019). Progress to Control and Eradication of Peste Petits Ruminants in the Southern African Development Community Region. Front. Vet. Sci..

[19] Jones B.A., Rich K.M., Mariner J.C., Anderson J., Jeggo M., Thevasagayam S., Cai Y., Peters A.R., Roeder P. (2016). The Economic Impact of Eradicating Peste Petits Ruminants: A Benefit-Cost Analysis. PLoS ONE.

[20] Tricco A.C., Lillie E., Zarin W., O’Brien K.K., Colquhoun H., Levac D., Moher D., Peters M.D.J., Horsley T., Weeks L. (2018). PRISMA Extension for Scoping Reviews (PRISMA-ScR): Checklist and Explanation. Ann. Intern. Med..

[21] Page M.J., McKenzie J.E., Bossuyt P.M., Boutron I., Hoffmann T.C., Mulrow C.D., Shamseer L., Tetzlaff J.M., Akl E.A., Brennan S.E. (2021). The PRISMA 2020 Statement: An Updated Guideline for Reporting Systematic Reviews. BMJ.

[22] Leboucq N., Ferrari G., Domenech J. (2015). PPR Monitoring and Evaluation Tool: A Companion Tool of the Global Strategy for the Control and Eradication of PPR.

[23] Food and Agriculture Organization of the United Nations Peste Petits Ruminants: Programme Approach. https://www.fao.org/ppr/global-programme/stepwise-approach/en/.

[24] Munir M. (2014). Role of Wild Small Ruminants in the Epidemiology of Peste Petits Ruminants. Transbound. Emerg. Dis..

[25] WOAH Sara Lysholm Update of Peste Petits Ruminants Global Control and Eradication Strategy (PPR GCES). Proceedings of the Presented at the WOAH Regional Workshop on PPR in Asia and the Pacific.

[26] FAO, WOAH (2022). Overview of The Plan of Action Peste Petits Ruminants Global Eradication Programme II & III Together for Peste Petits Ruminants Global Eradication by 2030 Blueprint 2022.

[27] Viola Chemis PPR Monitoring and Evaluation Tool Presented at the FMD/PPR Consultative meeting for East Mediterranean Countries, 2022. https://rr-middleeast.woah.org/app/uploads/2022/09/2-pmat-presentation-v-chemis.pdf.

[28] Dundon W.G., Diallo A., Cattoli G. (2020). Peste Petits Ruminants in Africa: A Review of Currently Available Molecular Epidemiological Data, 2020. Arch. Virol..

[29] AU-IBAR, FAO (2019). 7th Regional PPR Control And Eradication Coordination Committee (PPR-CECC) Meeting: The Communiqué.

[30] WOAH (2019). Burundi:Burundi—Peste Petits Ruminants Virus (Inf. with)—Follow up Report 8 [FINAL].

[31] Wendimu T.G., Dinbiso T.D., Lobago D.S., Marami L.M. (2024). Seroprevalence and Associated Risk Factors of Peste Petits Ruminants in Sheep and Goats in Three Districts of the Central Oromia Region, Ethiopia. Front. Vet. Sci..

[32] Fournié G., Waret-Szkuta A., Camacho A., Yigezu L.M., Pfeiffer D.U., Roger F. (2018). A Dynamic Model of Transmission and Elimination of Peste Petits Ruminants in Ethiopia. Proc. Natl. Acad. Sci. USA.

[33] Kumbe A., Negussie H., Getachew Y., Alemu B., Alemayehu G., Girma S., Sibhatu D., Emiyu K., Waktole H., Leta S. (2024). Epidemiology of Peste Petits Ruminants in Selected Districts of Borena Zone, Ethiopia. BMC Vet. Res..

[34] Nkamwesiga J., Coffin-Schmitt J., Ochwo S., Mwiine F.N., Palopoli A., Ndekezi C., Isingoma E., Nantima N., Nsamba P., Adiba R. (2019). Identification of Peste Petits Ruminants Transmission Hotspots in the Karamoja Subregion of Uganda for Targeting of Eradication Interventions. Front. Vet. Sci..

[35] Nkamwesiga J., Lumu P., Nalumenya D.P., Korennoy F., Roesel K., Wieland B., Perez A., Kiara H., Muhanguzi D. (2023). Seroprevalence and Risk Factors of Peste Petits Ruminants in Different Production Systems in Uganda. Prev. Vet. Med..

[36] Ayebazibwe C., Akwongo C.J., Waiswa J., Ssemakula O., Akandinda A., Barasa M., Nkamwesiga J., Mabirizi A., Lule P.M., Roesel K. (2022). Peste Petits Ruminants (PPR) in Uganda Assessment of Animal Health Systems and Coordination Mechanisms.

[37] Tounkara K. (2017). Western Africa Regional PPR Strategy and Roadmap. Presented at the OIE Procedures for the Endorsement of National Official Control Programmes with Regard to FMD and PPR.

[38] Adeoye F., Kolapo A., Ogunmolawa O., Aluko A., Meseko C., Oluwayelu D. (2022). Molecular Detection of Peste Petits Ruminants Virus (PPRV) in Goats and in Sheep in Ibadan, Oyo State, Nigeria. Vet. Ital..

[39] Tounkara K., Bataille A., Adombi C.M., Maikano I., Djibo G., Settypalli T.B.K., Loitsch A., Diallo A., Libeau G. (2018). First Genetic Characterization of Peste Petits Ruminants from Niger: On the Advancing Front of the Asian Virus Lineage. Transbound. Emerg. Dis..

[40] FAO, WOAH In Proceedings of the Atelier de Revue de la Feuille de Route Pour le Contrôle et L’éradication de la PPR dans les Pays de l’Union du Maghreb Arabe (UMA) une Approche Régionale Coordonnée.

[41] Intisar K.S., Ali Y.H., Haj M.A., Sahar M.A.T., Shaza M.M., Baraa A.M., Ishag O.M., Nouri Y.M., Taha K.M., Nada E.M. (2017). Peste Petits Ruminants Infection in Domestic Ruminants in Sudan. Trop. Anim. Health Prod..

[42] Ali S.E.M., Ahmed Y.A.M., Osman A.A., Gamal Eldin O.A., Osman N.A. (2023). Prevalence of Peste Petits Ruminants Virus Antibodies in Sheep and Goats Sera from Central-Western Sudan. Onderstepoort J. Vet. Res..

[43] Ali W.H., Osman N.A., Asil R.M., Mohamed B.A., Abdelgadir S.O., Mutwakil S.M., Mohamed N.E.B. (2019). Serological Investigations of Peste Petits Ruminants among Cattle in the Sudan. Trop. Anim. Health Prod..

[44] Soltan M.A., Abd-Eldaim M.M. (2014). Emergence of Peste Petits Ruminants Virus Lineage IV in Ismailia Province, Egypt. Infect. Genet. Evol. J. Mol. Epidemiol. Evol. Genet. Infect. Dis..

[45] WOAH (2022). Algeria—Peste Petits Ruminants Virus (Inf. with)—Follow up Report 3 [FINAL].

[46] (2004). Tunisia—Peste Petits Ruminants—Follow up Report 2. https://wahis.woah.org/#/in-review/2004.

[47] WAHIS Report: Morocco. https://wahis.woah.org/#/in-review/3991.

[48] Peste Petits Ruminants: Egypt. https://www.fao.org/ppr/current-situation/country-detail/en/?country_iso3=EGY.

[49] WAHIS Report: Libya. https://wahis.woah.org/#/in-event/3321/dashboard.

[50] Meetings of the West Eurasia PPR Roadmap (GF-TADs). https://rr-europe.woah.org/en/our-missions/animal-diseases/peste-des-petits-ruminants-ppr/ppr-west-eurasia-roadmap-meetings/#:~:text=2nd%20PPR%20Roadmap%20meeting,and%20vaccination%20in%20the%20region.

[51] World Organization for Animal Health Five Countries Receive Official Recognition of Animal Health Status from WOAH. https://www.woah.org/en/five-countries-receive-official-recognition-of-animal-health-status-from-woah/#:~:text=In%202024%2C%20five%20countries%20obtained,African%20horse%20sickness%20(AHS)%3B.

[52] Chenais E., Wennström P., Kartskhia N., Fischer K., Risatti G., Chaligava T., Enukidze T., Ståhl K., Vepkhvadze N.G. (2021). Perceptions of Pastoralist Problems: A Participatory Study on Animal Management, Disease Spectrum and Animal Health Priorities of Small Ruminant Pastoralists in Georgia. Prev. Vet. Med..

[53] FAO, WOAH In Proceedings of the Peste de Petits Ruminants Regional Meeting and Blueprint Consultation for Economic Cooperation Organization (ECO) Countries.

[54] Arede M., Beltrán-Alcrudo D., Aliyev J., Chaligava T., Keskin I., Markosyan T., Morozov D., Oste S., Pavlenko A., Ponea M. (2024). Corrigendum: Examination of Critical Factors Influencing Ruminant Disease Dynamics in the Black Sea Basin. Front. Vet. Sci..

[55] Kock R.A., Orynbayev M.B., Sultankulova K.T., Strochkov V.M., Omarova Z.D., Shalgynbayev E.K., Rametov N.M., Sansyzbay A.R., Parida S. (2015). Detection and Genetic Characterization of Lineage IV Peste Petits Ruminant Virus in Kazakhstan. Transbound. Emerg. Dis..

[56] Gao S., Xu G., Zeng Z., Lv J., Huang L., Wang H., Wang X. (2021). Transboundary Spread of Peste Petits Ruminants Virus in Western China: A Prediction Model. PLoS ONE.

[57] Abdrakhmanov S.K., Mukhanbetkaliyev Y.Y., Sultanov A.A., Yessembekova G.N., Borovikov S.N., Namet A., Abishov A.A., Perez A.M., Korennoy F.I. (2022). Mapping the Risks of the Spread of Peste Petits Ruminants in the Republic of Kazakhstan. Transbound. Emerg. Dis..

[58] Pruvot M., Fine A.E., Hollinger C., Strindberg S., Damdinjav B., Buuveibaatar B., Chimeddorj B., Bayandonoi G., Khishgee B., Sandag B. (2020). Outbreak of Peste Petits Ruminants among Critically Endangered Mongolian Saiga and Other Wild Ungulates, Mongolia, 2016–2017. Emerg. Infect. Dis..

[59] Benfield C.T.O., Hill S., Shatar M., Shiilegdamba E., Damdinjav B., Fine A., Willett B., Kock R., Bataille A. (2021). Molecular Epidemiology of Peste Petits Ruminants Virus Emergence in Critically Endangered Mongolian Saiga Antelope and Other Wild Ungulates. Virus Evol..

[60] Tounkara K., Kwiatek O., Niang M., Abou Kounta Sidibe C., Sery A., Dakouo M., Salami H., Lo M.M., Ba A., Diop M. (2019). Genetic Evidence for Transboundary Circulation of Peste Petits Ruminants Across West Africa. Front. Vet. Sci..

